# COVID‐19 Crisis Reduces Free Tropospheric Ozone Across the Northern Hemisphere

**DOI:** 10.1029/2020GL091987

**Published:** 2021-02-26

**Authors:** Wolfgang Steinbrecht, Dagmar Kubistin, Christian Plass‐Dülmer, Jonathan Davies, David W. Tarasick, Peter von der Gathen, Holger Deckelmann, Nis Jepsen, Rigel Kivi, Norrie Lyall, Matthias Palm, Justus Notholt, Bogumil Kois, Peter Oelsner, Marc Allaart, Ankie Piters, Michael Gill, Roeland Van Malderen, Andy W. Delcloo, Ralf Sussmann, Emmanuel Mahieu, Christian Servais, Gonzague Romanens, Rene Stübi, Gerard Ancellet, Sophie Godin‐Beekmann, Shoma Yamanouchi, Kimberly Strong, Bryan Johnson, Patrick Cullis, Irina Petropavlovskikh, James W. Hannigan, Jose‐Luis Hernandez, Ana Diaz Rodriguez, Tatsumi Nakano, Fernando Chouza, Thierry Leblanc, Carlos Torres, Omaira Garcia, Amelie N. Röhling, Matthias Schneider, Thomas Blumenstock, Matt Tully, Clare Paton‐Walsh, Nicholas Jones, Richard Querel, Susan Strahan, Ryan M. Stauffer, Anne M. Thompson, Antje Inness, Richard Engelen, Kai‐Lan Chang, Owen R. Cooper

**Affiliations:** ^1^ Deutscher Wetterdienst Hohenpeißenberg Germany; ^2^ Environment and Climate Change Canada Toronto ONT Canada; ^3^ Alfred Wegener Institut Helmholtz‐Zentrum für Polar‐ und Meeresforschung Potsdam Germany; ^4^ Danish Meteorological Institute Copenhagen Denmark; ^5^ Finnish Meteorological Institute Sodankylä Finland; ^6^ British Meteorological Service Lerwick UK; ^7^ University of Bremen Bremen Germany; ^8^ Institute of Meteorology and Water Management Legionowo Poland; ^9^ Deutscher Wetterdienst Lindenberg Germany; ^10^ Royal Netherlands Meteorological Institute DeBilt The Netherlands; ^11^ Met Éireann (Irish Met. Service) Valentia Ireland; ^12^ Royal Meteorological Institute of Belgium Uccle Belgium; ^13^ Karlsruhe Institute of Technology IMK‐IFU Garmisch‐Partenkirchen Germany; ^14^ Institute of Astrophysics and Geophysics University of Liège Liège Belgium; ^15^ Federal Office of Meteorology and Climatology MeteoSwiss Payerne Switzerland; ^16^ LATMOS Sorbonne Université‐UVSQ‐CNRS/INSU Paris France; ^17^ University of Toronto Toronto ONT Canada; ^18^ NOAA ESRL Global Monitoring Laboratory Boulder CO USA; ^19^ Cooperative Institute for Research in Environmental Sciences (CIRES) University of Colorado Boulder CO USA; ^20^ National Center for Atmospheric Research Boulder CO USA; ^21^ State Meteorological Agency (AEMET) Madrid Spain; ^22^ Meteorological Research Institute Tsukuba Japan; ^23^ Jet Propulsion Laboratory California Institute of Technology Table Mountain Facility Wrightwood CA USA; ^24^ Izaña Atmospheric Research Center AEMET Tenerife Spain; ^25^ Karlsruhe Institute of Technology IMK‐ASF Karlsruhe Germany; ^26^ Bureau of Meteorology Melbourne Australia; ^27^ Centre for Atmospheric Chemistry University of Wollongong Wollongong Australia; ^28^ National Institute of Water and Atmospheric Research Lauder New Zealand; ^29^ NASA Goddard Space Flight Center Earth Sciences Division Greenbelt MD USA; ^30^ Universities Space Research Association Columbia MD USA; ^31^ Earth System Science Interdisciplinary Center University of Maryland College Park MD USA; ^32^ European Centre for Medium‐Range Weather Forecasts Reading UK; ^33^ NOAA Chemical Sciences Laboratory Boulder CO USA

**Keywords:** COVID‐19, emissions, ozone, troposphere

## Abstract

Throughout spring and summer 2020, ozone stations in the northern extratropics recorded unusually low ozone in the free troposphere. From April to August, and from 1 to 8 kilometers altitude, ozone was on average 7% (≈4 nmol/mol) below the 2000–2020 climatological mean. Such low ozone, over several months, and at so many stations, has not been observed in any previous year since at least 2000. Atmospheric composition analyses from the Copernicus Atmosphere Monitoring Service and simulations from the NASA GMI model indicate that the large 2020 springtime ozone depletion in the Arctic stratosphere contributed less than one‐quarter of the observed tropospheric anomaly. The observed anomaly is consistent with recent chemistry‐climate model simulations, which assume emissions reductions similar to those caused by the COVID‐19 crisis. COVID‐19 related emissions reductions appear to be the major cause for the observed reduced free tropospheric ozone in 2020.

## Key Points

- In spring and summer 2020, stations in the northern extratropics report on average 7% (4 nmol/mol) less tropospheric ozone than normal
- Such low tropospheric ozone, over several months, and at so many sites, has not been observed in any previous year since at least 2000
- Most of the reduction in tropospheric ozone in 2020 is likely due to emissions reductions related to the COVID‐19 pandemic

## 1 Introduction

Widespread measures to contain the COVID‐19 pandemic have slowed, or even closed down, industries, businesses, and transportation activities, and have reduced anthropogenic emissions substantially throughout the year 2020. Guevara et al. (2020), or Barré et al. (2020) report European emissions reductions up to 60% for NO_*x*_, and up to 15% for Non‐Methane Volatile Organic Compounds (NMVOC) in March/April 2020. Based on satellite observations of NO_2_ columns (Bouwens et al., 2020), comparable NO_x_ emissions reductions are reported for Chinese cities in February 2020 (Ding et al., 2020; Feng et al., 2020). Globally averaged CO_2_ emissions decreased by 8.8% during the first half of 2020 (Z. Liu et al., 2020), consistent in timing and magnitude with the aforementioned NO_2_ emission reductions. The largest relative reductions occurred for air traffic, where emissions decreased by ≈40%, on average, in the first half of 2020 (Le Quéré et al., 2020a; Z. Liu et al., 2020), and remained low during the second half of 2020 (Le Quéré et al., 2020b).

These COVID‐19 emissions reductions are large enough to affect ozone levels in the troposphere (Dentener et al., 2011). Tropospheric O_3_‐NO_*x*_‐VOC‐HO_x_ chemistry is, however, complex and nonlinear. The net effect of emission changes depends on NO_*x*_ and VOC concentrations (e.g., Kroll et al., 2020; Sillman, 1999; Thornton et al., 2002). In polluted regions, at high NO_*x*_ concentrations (>> 1pbb), reducing NO_*x*_ concentrations can increase ozone, because ozone titration by NO is reduced (e.g., Sicard et al., 2020). At low concentrations (NO_*x*_ < 1 nmol/mol), however, in the clean or mildly polluted free troposphere, reducing NO_x_ lowers photochemical ozone production (e.g., Bozem et al., 2017), and results in less ozone.

Indeed, for many polluted regions, studies report increased near‐surface ozone after COVID‐19 lockdowns (e.g., Collivignarelli et al., 2020; Lee et al., 2020; Shi & Brasseur, 2020; Siciliano et al., 2020; Venter et al., 2020). Reduced surface ozone is reported for some rural areas, e.g., in the US and Western Europe (Chen et al., 2020; Menut et al., 2020). Meteorological conditions complicate matters, as they play an important role as well (Goldberg et al., 2020; Keller et al., 2021; Ordóñez et al., 2020; Shi & Brasseur, 2020).

In the free troposphere, ozone is an important greenhouse gas, and plays a key role in tropospheric chemical reactions, controlling the oxidizing capacity (e.g. Archibald et al., 2020; Cooper et al., 2014; Gaudel et al, 2018). The Northern Hemisphere free troposphere is dominated by net photochemical ozone production, proportional (albeit nonlinearly) to the availability of ozone precursor gases (e.g., Zhang et al., 2020). In contrast to increases of surface ozone in polluted urban areas after the COVID‐19 emissions reductions, we find significant reductions of ozone in the northern extratropical free troposphere. These large‐scale reductions occurred in late spring and summer 2020, following the widespread COVID‐19 slowdowns, and are unique within the last two decades.

## 2 Instruments and Data

Regular observations of ozone in the free troposphere are sparse: Only around 50 ozone sounding stations worldwide (e.g. Tarasick et al., 2019), a handful of tropospheric LIDARs (Gaudel et al., 2015; Leblanc et al., 2018), and about twenty Fourier Transform Infrared Spectrometers (FTIRs, Vigouroux et al., 2015). In‐Service Aircraft for a Global Observing System (IAGOS, Nédélec et al., 2015) are another important source of tropospheric ozone data. Due to the COVID‐19 slowdowns, however, few IAGOS aircraft were flying in 2020, and IAGOS data became quite sparse, with only about 20 flights per month since April 2020, compared to more than 200 flights per month in 2019. The information content of satellite measurements on ozone in the free troposphere is limited, and accuracy is modest, 10%–30% (Hurtmans et al., 2012; Liu et al., 2010; Oetjen et al., 2014). The recent Tropospheric Ozone Assessment Report found large differences in tropospheric ozone trends derived from different satellite instruments, and even different signs in some regions (Gaudel et al., 2018).

Ozonesondes measure profiles with high vertical resolution, about 100 m, and good accuracy, 5%–15% in the troposphere, 5% in the stratosphere (Smit et al., 2007; Sterling et al., 2018; Tarasick et al., 2016; Van Malderen et al., 2016; Witte et al., 2017; WMO, 2014). This is adequate to detect ozone anomalies of several percent. We use stations with regular soundings, at least once per month since the year 2000, and with data available until at least July 2020. Soundings with obvious deficiencies were rejected (i.e. large data gaps, integrated ozone column from the sounding deviating by more than 30% from ground‐ or satellite‐based spectrometer measurement). Table 1 provides information on stations, and public data archives.

**Table 1:** Stations in This Study, Mostly Ozonesonde Stations

Station	Latitude ( N)	Longitude (E)	Data source (see caption)	Data until	Profiles/spectra per month in 2020
Alert, Canada^a^ ^,^ ^c^	82.50	−62.34	W	4/2020	3.75
Eureka, Canada^c^	80.05	−86.42	W, E	9/2020	4.89
Ny‐Ålesund, Norway	78.92	11.92	W, E	10/2020	7.10
Ny‐Ålesund FTIR, Norway	78.92	11.92	N	7/2020	12.86
Thule FTIR, Greenland	76.53	−68.74	N	9/2020	73
Resolute, Canada^a^	74.72	−94.98	W	4/2020	5.50
Scoresbysund, Greenland	70.48	−21.95	E	11/2020	4.00
Kiruna FTIR, Sweden	67.41	20.41	N	7/2020	46
Sodankylä, Finland	67.36	26.63	W, E	12/2020	2.83
Lerwick, United Kingdom	60.13	−1.18	W, E	12/2020	3.92
Churchill, Canada ^a, c^	58.74	−93.82	W	3/2020	3.33
Edmonton, Canada ^a^ ^,^ ^c^	53.55	−114.10	W	3/2020	3.67
Goose Bay, Canada ^a^	53.29	−60.39	W	3/2020	2.67
Bremen FTIR, Germany	53.13	8.85	N	10/2020	5.27
Legionowo, Poland	52.40	20.97	W	10/2020	4.00
Lindenberg, Germany	52.22	14.12	W	11/2020	4.73
DeBilt, Netherlands	52.10	5.18	W, E	12/2020	4.33
Valentia, Ireland	51.94	−10.25	W, E	12/2020	2.50
Uccle, Belgium	50.80	4.36	W, E	12/2020	12.00
Hohenpeissenberg, Germany	47.80	11.01	W	12/2020	10.50
Zugspitze FTIR, Germany	47.42	10.98	N	9/2020	73
Jungfraujoch FTIR, Switzerland	46.55	7.98	N	12/2020	46
Payerne, Switzerland	46.81	6.94	W	10/2020	11.10
Haute Provence, France	43.92	5.71	N	8/2020	2.50
Haute Provence LIDAR, France	43.92	5.71	N	8/2020	3.50
Toronto FTIR, Canada	43.66	−79.40	N	10/2020	59
Trinidad Head, California, USA	41.05	−124.15	G	12/2020	3.58
Madrid, Spain	40.45	−3.72	W	11/2020	4.09
Boulder, Colorado, USA	39.99	−105.26	G	12/2020	4.83
Boulder FTIR, Colorado, USA	39.99	−105.26	N	10/2020	56
Tateno (Tsukuba), Japan ^b^	36.05	140.13	W	10/2020	2.70
Table Mountain LIDAR, California, USA	34.40	−117.70	N	8/2020	19
Izana, Tenerife, Spain	28.41	−16.53	W	8/2020	2.00
Izana FTIR, Tenerife, Spain	28.30	−16.48	N	9/2020	28
Hong Kong, China	22.31	114.17	W	9/2020	4.11
Hilo, Hawaii, USA ^c^	19.72	−155.07	G	12/2020	4.08
Mauna Loa FTIR, Hawaii, USA	19.54	−155.58	N	10/2020	36
Paramaribo, Suriname	5.81	−55.21	N, E	10/2020	3.60
Pago Pago, American Samoa ^c^	−14.25	−170.56	G	12/2020	3.08
Suva, Fiji ^c^	−18.13	178.32	G	9/2020	1.44
Wollongong FTIR, Australia	−34.41	150.88	N	10/2020	43
Broadmeadows, Australia	−37.69	144.95	W	7/2020	4.29
Lauder, New Zealand	−45.04	169.68	W	10/2020	4.40
Lauder FTIR, New Zealand	−45.04	169.68	N	10/2020	99
Macquarie Island, Australia	−54.50	158.94	W	7/2020	4.29

Data sources: W = World Ozone and UV Data Centre (https://woudc.org/archive/Archive‐NewFormat/OzoneSonde_1.0_1/), N = Network for the Detection of Atmospheric Composition Change (ftp://ftp.cpc.ncep.noaa.gov/ndacc/station/; ftp://ftp.cpc.ncep.noaa.gov/ndacc/RD/), E = European Space Agency Validation Data Center (https://evdc.esa.int/ requires registration, or ftp://zardoz.nilu.no/nadir/projects/vintersol/data/o3sondes requires account), G = Global Monitoring Laboratory, National Oceanic and Atmospheric Administration (ftp://aftp.cmdl.noaa.gov/data/ozwv/Ozonesonde/). FTIR, Fourier Transform Infrared Spectrometers.

^a^ Due to COVID‐19 restrictions, most Canadian ozonesonde data were available only up to March or April 2020.

^b^ Tateno data were corrected for the change from Carbon Iodine to ECC ozonesondes in December 2009.

^c^ Stations affected by a drop‐off in ECC sonde sensitivity >3% in the stratosphere, after 2015 (see Stauffer et al., 2020). The drop‐off is much smaller (<<1%) in the troposphere, and should be negligible here. At many of the affected stations, ECC sondes behaved normally again in 2019/2020.

Apart from the sondes, FTIR spectrometers from the Network for the Detection of Atmospheric Composition Change (NDACC, De Mazière et al., 2018) provide independent information, based on a completely different method (ground‐based solar‐infrared absorption spectrometry). The altitude resolution of FTIR ozone profiles in the troposphere is much coarser (5–10 km) than that of the sondes, while accuracy is similar, 5%–10% (Vigouroux et al., 2015). Finally, we use data from tropospheric lidars (Gaudel et al., 2015; Granados‐Muñoz & Leblanc, 2016), which provide ozone profiles from ≈3 to 12 km altitude, with accuracy comparable to the sondes (5%–10%; Leblanc et al., 2018), and slightly coarser altitude resolution (100 m–2 km).

We also use global atmospheric composition re‐analyses from the Copernicus Atmosphere Monitoring Service for the years 2003–2019, and operational analyses for the year 2020 (CAMS, Inness et al., 2019; see also Park et al., 2020). The CAMS data are taken at the grid‐points closest to the stations in Table 1. The analyses (in 2020) are adjusted for the small average difference to the re‐analyses in 2018 and 2019. CAMS (re‐)analyses are based on meteorological fields, and assimilation of satellite observations of ozone and NO_2_. However, for NO_2_ the impact of the assimilation is small and frequently insignificant, so that tropospheric NO_x_ in CAMS is essentially controlled by the prescribed emissions (Inness et al., 2019). Similarly, the limited information content of current satellite measurements of tropospheric ozone means that tropospheric ozone in CAMS is also driven largely by the prescribed emissions (and the chemistry module). Stratospheric ozone, however, is constrained well by the assimilated satellite data. Thus, CAMS analyses account for the large Arctic stratospheric depletion in spring of 2020 (Manney et al., 2020; Wohltmann et al., 2020), for 2020 meteorological conditions, and for ozone transport, e.g. from the stratosphere to the troposphere (Neu et al., 2014). However, since they rely on “business as usual” emissions for 2020, the CAMS analyses do not account for the effects of COVID‐19 emissions reductions in 2020 on tropospheric ozone (and NO_x_).

## 3 Results

**Figure 1:**
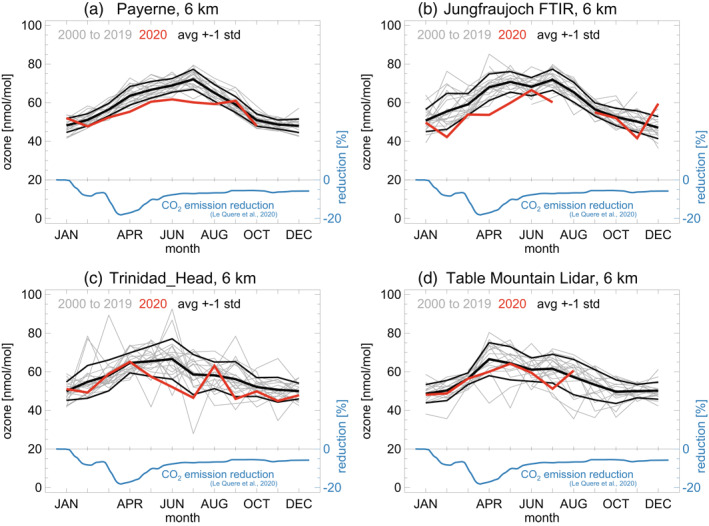
Observed ozone monthly means at four typical stations. Results are for 6 km altitude. The thick red line highlights the year 2020. Climatological averages, and standard deviations over the years 2000–2020 are indicated by the thick black lines. Payerne (a) and Trinidad Head (c) are sonde stations. Jungfraujoch (b) is an FTIR station. Table Mountain (d) is a lidar station. Dark blue lines and scale on the right: CO_2_ emission reduction (in percent) from Le Quéré et al. (2020b), as a proxy for ozone precursor reductions in 2020. FTIR, Fourier Transform Infrared Spectrometer.

For selected stations, Figure 1 presents the annual cycles of tropospheric ozone over the last 20 years, at 6 km, a representative altitude for the free troposphere. Monthly means (over 1 km wide layers) reduce synoptic meteorological variability and measurement noise, and focus on longer‐term, larger‐scale variations.

Payerne, Jungfraujoch, and Trinidad Head show an annual cycle with low ozone in winter and high ozone in summer. This is the case for most stations in the northern extratropics (Cooper et al., 2014; Gaudel et al., 2018; Parrish et al., 2020). Increased photochemical production due to more sunlight and warmer temperatures is the main driver for the summer ozone maximum in the northern extratropics (Archibald et al., 2020; Wu et al., 2007).

Figure 1 shows substantial yearly variability, but ozone levels are notably below average in 2020, at all four stations (thick red lines in Figure 1). At Payerne and Jungfraujoch, and a number of other stations, monthly means in spring and summer 2020 were actually the lowest, or close to the lowest, since 2000. For context, the dark blue lines in Figure 1 provide global CO_2_ emission reductions due to the COVD‐19 pandemic (Le Quéré et al., 2020b). Comparable reductions apply to global ozone precursor emissions (NO_x_ and VOCs). The (daily) emission reductions in Figure 1 indicate that the largest effect for ozone might be expected after March 2020. However, Figure 1 does not show any clear or close correspondence between unusual ozone monthly means in 2020 (red lines) and the emission reductions (dark blue lines).

**Figure 2:**
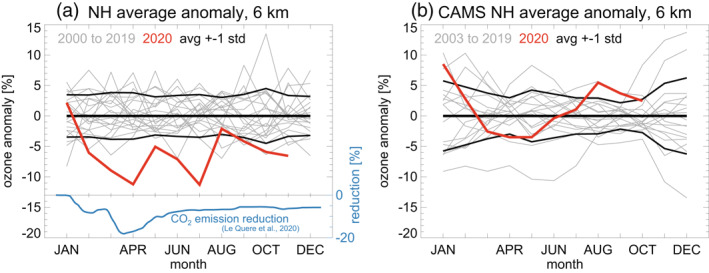
Annual cycles of monthly mean northern extratropical ozone anomalies at 6 km altitude. Anomalies are in percent, relative to the climatological monthly mean calculated for each station/ instrument, and for the period 2000–2020 (all Januaries, all Februaries, …, all Decembers). These single station/instrument anomalies are then averaged over all northern extratropical stations/instruments (north of 15°N). Panel (a) Results from the station observations. Panel (b) Results for CAMS atmospheric composition (re‐)analyses at grid points nearest the stations. The CAMS data do not account for COVID‐19 related emissions reductions in 2020. Gray lines: individual years from 2000 to 2019. Thick red line: year 2020. Thick black lines: average anomaly, ±1 standard deviation over the years. Dark blue lines and scale on the right in panel (a): Global CO_2_ emission reduction in 2020 (in percent) from Le Quéré et al. (2020b), as in Figure 1. CAMS, Copernicus Atmosphere Monitoring Service.

Annual cycles of ozone anomalies, averaged over all northern extratropical stations (stations north of 15°N), are shown in Figure 2. Anomalies were defined as the relative deviation (in percent) from the 2000 to 2020 climatological mean of each calendar month at each station. As for the single stations in Figure 1, the observed northern extratropical average shows exceptionally low ozone throughout spring and summer 2020 (red line in Figure 2a). This is not reproduced by the CAMS analyses, which do not account for COVID‐19 related emissions reductions, and simulate ozone in the usual range in 2020 (red line in Figure 2b). Again, there is no close temporal correspondence between the unusual behavior of observed ozone in 2020 (red line in Figure 2a), and the emission reductions (dark blue line in Figure 2a).

**Figure 3:**
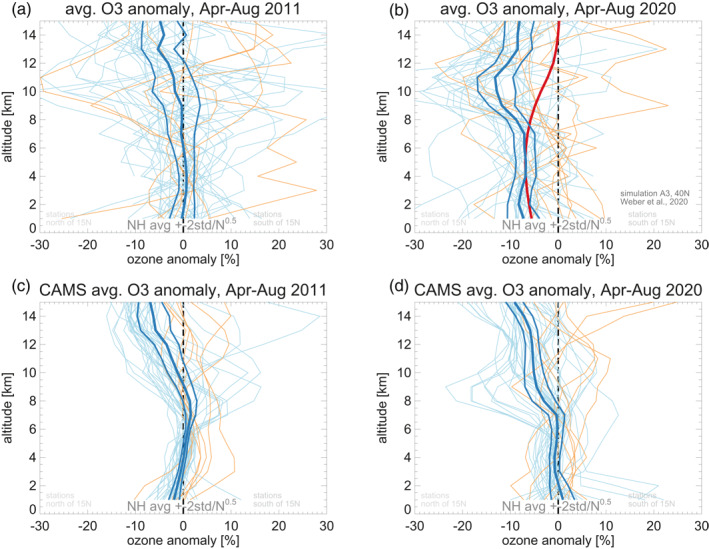
Ozone anomaly profiles (in percent), averaged over April to August. Stations are excluded in years where their data cover less than three of these five months. Panel (a) for the year 2011. Panel (b) for the year 2020. Light blue lines: northern extratropical stations (north of 15°N). Light orange lines: remaining stations, south of 15°N. Thick dark blue line: mean of the northern extratropical stations. Thin dark blue lines: 95% confidence interval of the mean of the northern extratropical stations. Red line in panel (b): simulated ozone change at 40°N from Weber et al. (2020; Figure S4, scenario A3). Panels (c), (d): Same as (a), (b), but for CAMS (re‐)analyses at the grid‐points closest to the stations. CAMS, Copernicus Atmosphere Monitoring Service.

Figures 1 and 2 show large negative anomalies from April to August 2020. Figure 3 compares anomaly profiles averaged over those five calendar months, between the years 2011 and 2020. Both years saw unusually large springtime ozone depletion in the Arctic stratosphere (Manney et al., 2020; Wohltmann et al., 2020). In the stratosphere, above ≈10 km, the Arctic depletion appears as low ozone, both in observations and CAMS results (particularly for stations north of 50°N). In both the stratosphere and the troposphere, the observed profiles show more variability than the smoother CAMS profiles. In 2020, most observed single station anomaly profiles (Figure 3b) are negative throughout the northern extratropical troposphere (between 1 and 10 km). This is not the case in 2011 (Figures 3a and 3c), nor in the CAMS data in 2020 (Figure 3d).

The 2020 anomaly is even clearer for the northern extratropical mean profile (dark blue lines in Figure 3). The observed 2020 mean anomaly profile is large, −6% to −9%, and statistically significant at the 95% level (more than 99% in fact) from 1 to 8 km (Figure 3b), whereas the corresponding CAMS profile is close to zero (Figure 3d). Figure 3 indicates that Arctic stratospheric springtime ozone depletion did not have a large effect on tropospheric ozone below 8 km in 2011 and 2020 (see also model simulations in Figure S1, based on Gelaro et al., 2017, and Strahan et al., 2019), and that the CAMS “business as usual” simulation does not account for the observed large negative tropospheric anomaly in 2020.

Figure 3b also shows a simulated profile of tropospheric ozone reduction from a recent chemistry‐climate modeling study of COVID‐like emissions decreases by Weber et al. (2020). This simulated profile (red line in our Figure 3b) matches the observed northern extratropical ozone reduction (dark blue line), from the ground up to about 8 km. Above 8 km, the simulated profile deviates by ≈10% from the observed profile, because it assumes fixed 2012 to 2014 meteorological conditions. The CAMS analyses (Figure 3d) show that 2020 meteorological conditions and springtime Arctic stratospheric ozone depletion resulted in ozone reductions of 5%–10% above 9 km, consistent with the observations.

**Figure 4:**
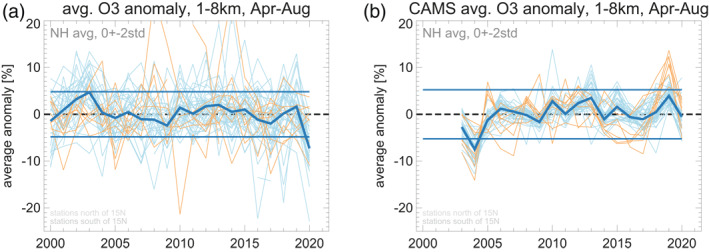
Tropospheric ozone anomaly, averaged over April to August and from 1 to 8 km, for the years 2000–2020. Panel (a) Observations. Panel (b) CAMS atmospheric composition (re‐)analyses. Light blue lines: northern extratropical stations (north of 15°N). Light orange lines: stations south of 15°N. Thick dark blue line: Average over all stations north of 15°N. Thin dark blue lines: ±2 standard deviations over all years of this average. CAMS, Copernicus Atmosphere Monitoring Service.

Time series of the tropospheric anomaly (averaged from April to August, and from 1 to 8 km altitude) are shown in Figure 4. In the observations (left panel), the year 2020 stands out with large negative anomalies, not seen in the CAMS data. Across the 20 previous years, ozone anomalies at individual stations (thin lines) are scattered around zero. The northern extratropical average anomaly (dark blue line) is usually smaller than ±3%. The only other observed exception is the positive anomaly related to the (European) heat‐wave summer of 2003 (Vautard et al., 2007). The large negative northern extratropical anomaly in the observations in 2020, ≈−7%, is clearly outside of the ±2σ range of the previous 20 years (thin dark blue lines). It is not reproduced by the CAMS “emissions as usual” analysis.

**Figure 5:**
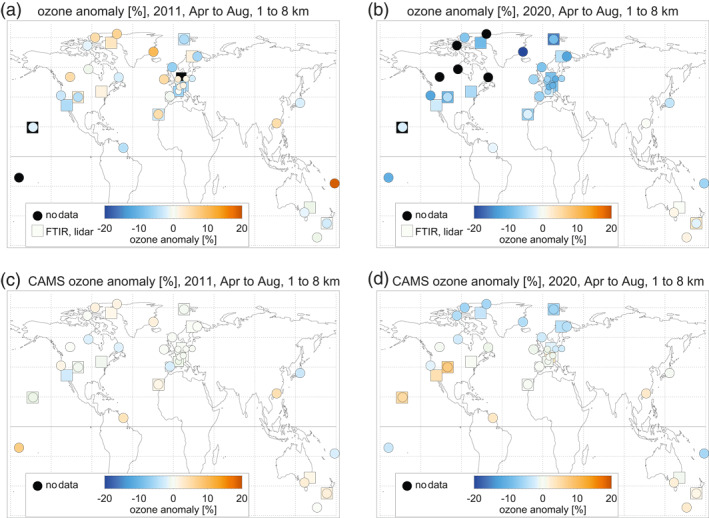
Geographic distribution of observed tropospheric ozone anomalies (averaged over the months April to August, and over altitudes from 1 to 8 km) for the years (a) 2011 and (b) 2020. Panels (c) and (d): same, but for CAMS results at the station locations. Colored circles give the anomaly at the ozonesonde stations. Squares are for FTIR and lidar stations. See Table S1 of the supplement for the numerical values. Black filling indicates insufficient data in the given year. CAMS, Copernicus Atmosphere Monitoring Service; FTIR, Fourier Transform Infrared Spectrometers.

The geographic distribution of the average tropospheric ozone anomalies is shown for 2011 and 2020 in Figure 5. 2020 stands out in the observations with large negative anomalies at nearly all northern extratropical stations, and a fairly uniform geographical distribution (see Table S1 of the supplement for the numerical values). CAMS does show negative anomalies in 2020, but only north of 50°N, and not as large as the observations. In the Southern Hemisphere in 2020, agreement between observations and CAMS is quite good, typically within 2.5% or better (see also Table S1). In 2011, some stations show positive anomalies, negative anomalies are not as large as in 2020, and the geographical distribution is less uniform. Agreement between observations and CAMS is reasonable in 2011, usually within a few percent.

## 4 Discussion and Conclusions

Ozone stations in the northern extratropics indicate exceptionally low ozone in the free troposphere (1–8 km) in spring and summer 2020. Compared to the 2000–2020 climatology, ozone was reduced by 7% (≈4 nmol/mol). Such widespread low tropospheric ozone, across so many stations and over several months has not been observed in any previous year since 2000. The observed 7% ozone reduction in the free troposphere stands in contrast to increases of surface ozone by 10%–30%, reported for many polluted urban areas after the COVID‐19 related emissions reductions in 2020 (e.g., Collivignarelli et al., 2020; Lee et al., 2020; Shi & Brasseur, 2020; Siciliano et al., 2020; Venter et al., 2020). However, the chemical regime for ozone in the free troposphere is different (e.g., Kroll et al., 2020; Sillman, 1999; Thornton et al., 2002), and free tropospheric ozone reductions are expected after the substantial decrease of precursor emissions due to the COVID‐19 pandemic (e.g. Guevara et al., 2020; Zhang et al., 2020).

Recent model simulations of COVID‐like emissions decreases (Weber et al., 2020) find tropospheric ozone reductions very similar to our observational results. From our results, and the simulations by Weber et al., 2020, it appears that the total tropospheric ozone burden of the northern extratropics decreased by about 7% for April–August 2020. The contribution from ozone increases in polluted urban areas to the total burden is opposite, but very small.

The Weber et al. (2020) simulations indicate that the major causes of tropospheric ozone reduction come from reduced surface transportation (ozone decrease throughout most of the northern extratropical troposphere), and from reduced aviation (ozone decrease mostly between 10 and 12 km altitude and north of 30°N, see also Grewe et al., 2017). While the simulations are qualitatively consistent with the observations, they consider only March to May. New simulations using more recent and extended emissions estimates (Le Quéré et al., 2020b), and further comparison with our station observations would be worthwhile.

The observed large and fairly uniform 7% reduction of ozone in the northern extratropical troposphere in spring and summer 2020 provides a far reaching test case for the response of tropospheric ozone to emission changes. Further quantification of this anomaly will be possible, when observations from commercial aircraft (IAGOS), and satellite instruments become available. Additional modeling studies will improve our understanding of the contributions from different sectors such as air traffic, and surface transportation.

## Supporting information

Supporting Information S1

Figure S1

## Data Availability

Most of the ozonesonde data used in this study are freely available from the World Ozone and UV Data Center (https://woudc.org) at Environment Canada (https://exp‐studies.tor.ec.gc.ca/), and are downloadable at https://woudc.org/archive/Archive‐NewFormat/OzoneSonde_1.0_1/). Some ozonesonde data for 2020 were not yet available at the WOUDC. Instead, rapid delivery data were obtained from ftp://zardoz.nilu.no/nadir/projects/vintersol/data/o3sondes (requires registration), at the Nadir database of the Norwegian Institute for Air Quality (NILU, https://projects.nilu.no/nadir/obs.html). Registration information, and the same data in a different format, are available from the European Space Agency Validation Data Center (https://evdc.esa.int/). For Boulder, Trinidad Head, Hilo, Fiji, and Samoa, stations operated by the US National Oceanic and Atmospheric Administration, Global Monitoring Laboratory (https://www.esrl.noaa.gov/gmd/ozwv/), data can be obtained freely from ftp://aftp.cmdl.noaa.gov/data/ozwv/Ozonesonde/. FTIR and lidar data, as well as some ozonesonde data, are from the Network for the Detection of Atmospheric Composition Change (https://ndacc.org), and are freely available at ftp://ftp.cpc.ncep.noaa.gov/ndacc/station/ and ftp://ftp.cpc.ncep.noaa.gov/ndacc/RD/. Copernicus Atmosphere Monitoring Service (CAMS) global chemical weather EAC4 re‐analyses are available at https://atmosphere.copernicus.eu/data. CAMS operational global analyses and forecasts are available at https://apps.ecmwf.int/datasets/data/cams‐nrealtime/.
