# Supplementary material for: COVID‐19 Crisis Reduces Free Tropospheric Ozone Across the Northern Hemisphere
Source: Geophys Res Lett. 2021 Feb 26;48(5):e2020GL091987. doi: 10.1029/2020GL091987 (PMC7995013; doi:10.1029/2020GL091987)
Supplement: Supplementary file 1 — Supporting Information S1 [file GRL-48-e2020GL091987-s001.docx]

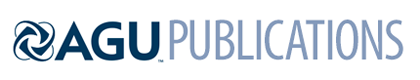


*Geophyscial Research Letters*

Supporting Information for

**COVID-19 Crisis Reduces Free Tropospheric Ozone across the Northern Hemisphere**

Wolfgang Steinbrecht^1^, Dagmar Kubistin^1^, Christian Plass-Dülmer^1^, Jonathan Davies^2^, David W. Tarasick^2^, Peter von der Gathen^3^, Holger Deckelmann^3^, Nis Jepsen^4^, Rigel Kivi^5^, Norrie Lyall^6^, Matthias Palm^7^, Justus Notholt^7^, Bogumil Kois^8^, Peter Oelsner^9^, Marc Allaart^10^, Ankie Piters^10^, Michael Gill^11^, Roeland Van Malderen^12^, Andy W. Delcloo^12^, Ralf Sussmann^13^, Emmanuel Mahieu^14^, Christian Servais^14^, Gonzague Romanens^15^, Rene Stübi^15^, Gerard Ancellet^16^, Sophie Godin-Beekmann^16^, Shoma Yamanouchi^17^, Kim Strong^17^, Bryan Johnson^18^, Patrick Cullis^18, 19^, Irina Petropavlovskikh^18, 19^, James W. Hannigan^20^, Jose-Luis Hernandez^21^, Ana Diaz Rodriguez^21^, Tatsumi Nakano^22^, Fernando Chouza^23^, Thierry Leblanc^23^, Carlos Torres^24^, Omaira Garcia^24^, Amelie N. Röhling^25^, Matthias Schneider^25^, Thomas Blumenstock^25^, Matt Tully^26^, Clare Paton-Walsh^27^, Nicholas Jones^27^, Richard Querel^28^, Susan Strahan^29,30^, Ryan M. Stauffer^29,33^, Anne M. Thompson^29^, Antje Inness^31^, Richard Engelen^31^, Kai-Lan Chang^32,19^, Owen R. Cooper^32,19^

^1^Deutscher Wetterdienst, Hohenpeißenberg, Germany.

^2^Environment and Climate Change Canada, Toronto, Canada.

^3^Alfred Wegener Institut, Helmholtz-Zentrum für Polar- und Meeresforschung, Potsdam, Germany.

^4^Danish Meteorological Institute, Copenhagen, Denmark.

^5^Finnish Meteorological Institute, Sodankylä, Finland.

^6^British Meteorological Service, Lerwick, United Kingdom.

^7^University of Bremen, Bremen, Germany.

^8^Institute of Meteorology and Water Management, Legionowo, Poland.

^9^Deutscher Wetterdienst, Lindenberg, Germany.

^10^Royal Netherlands Meteorological Institute, DeBilt, The Netherlands.

^11^Met Éireann (Irish Met. Service), Valentia, Ireland.

^12^Royal Meteorological Institute of Belgium, Uccle, Belgium.

^13^Karlsruhe Institute of Technology, IMK-IFU, Garmisch-Partenkirchen, Germany.

^14^Institute of Astrophysics and Geophysics, University of Liège, Liège, Belgium.

^15^Federal Office of Meteorology and Climatology, MeteoSwiss, Payerne, Switzerland.

^16^LATMOS, Sorbonne Université-UVSQ-CNRS/INSU, Paris, France.

^17^University of Toronto, Toronto, Canada.

^18^NOAA ESRL Global Monitoring Laboratory, Boulder, CO, USA.

^19^Cooperative Institute for Research in Environmental Sciences (CIRES), University of Colorado, Boulder, CO, USA.

^20^National Center for Atmospheric Research, Boulder, CO, USA.

^21^State Meteorological Agency (AEMET), Madrid, Spain.

^22^Meteorological Research Institute, Tsukuba, Japan.

^23^ Jet Propulsion Laboratory, California Institute of Technology, Table Mountain Facility, Wrightwood, CA, USA.

^24^Izaña Atmospheric Research Center, AEMET, Tenerife, Spain.

^25^Karlsruhe Institute of Technology, IMK-ASF, Karlsruhe, Germany.

^26^Bureau of Meteorology, Melbourne, Australia.

^27^Centre for Atmospheric Chemistry, University of Wollongong, Wollongong, Australia.

^28^National Institute of Water and Atmospheric Research, Lauder, New Zealand.

^29^Earth Sciences Division, NASA Goddard Space Flight Center, Greenbelt, MD, USA.

^30^Universities Space Research Association, Columbia, MD, USA.

^31^European Centre for Medium-Range Weather Forecasts, Reading, United Kingdom.

^32^NOAA Chemical Sciences Laboratory, Boulder, CO, USA.

^33^Earth System Science Interdisciplinary Center, University of Maryland, College Park, MD, USA

Corresponding author: Wolfgang Steinbrecht ([wolfgang.steinbrecht@dwd.de](mailto:wolfgang.steinbrecht@dwd.de))

**Contents of this file**

Text S1

Figure S1

Table S1

**Introduction**

The supplementary material presented here gives additional information on:

- the magnitude of tropospheric ozone reductions that may have been caused by the large springtime ozone depletion of the Arctic stratosphere in 2020.
- the numerical values of the average tropospheric ozone reduction observed in 2020 at the individual stations, and simulated by CAMS at the closest gridpoints.

Text S1.

Figure S1 shows the difference between two simulations by the Global Modeling Initiative (GMI) chemistry transport model (Strahan et al., 2019), based on meteorological fields from MERRA2 re-analysis (Gelaro et al., 2017). One simulation includes the large Arctic ozone depletion caused in spring 2020 by heterogeneous chemistry in the polar vortex; the other simulation does not. The difference between the two simulations provides an estimate for the effect of 2020 Arctic stratospheric depletion on ozone in the troposphere. According to the simulations, the tropospheric effect is similar at most latitudes north of 40° to 50°N. It is smaller than 1 ppbv (or ≈2%) on average, and is largest in June 2020.

Figure S1. Latitude - altitude cross sections of tropospheric ozone reductions (in nmol/mol), attributed to the large Arctic springtime stratospheric ozone depletion of 2020. Latitudes from 20°N to 90°N, altitudes from 0 km to 8 km. Top panel is for March 1^st^, middle panel for June 1^st^, bottom panel for August 28^th^. Results are from two simulations by the Global Modeling Initiative (GMI) chemistry transport model (Strahan et al., 2019), based on meteorological fields from the MERRA2 re-analysis (Gelaro et al., 2017). One simulation includes ozone depletion caused by heterogeneous chemistry in the Arctic polar vortex. The other simulation does not. The plotted difference gives an estimate of how much the large Arctic stratospheric ozone depletion in spring 2020 contributed to reduced ozone in the troposphere.

| **Station** | **Latitude (deg N)** | **Longitude (deg E)** | **observed average anomaly 2020 [%]** | **CAMS average anomaly 2020 [%]** |
| --- | --- | --- | --- | --- |
| Alert, Canada | 82.50 | -62.34 | N/A | -5.5 |
| Eureka, Canada | 80.05 | -86.42 | -7.8 | -5.8 |
| Ny-Ålesund, Norway | 78.92 | 11.92 | -8.8 | -5.5 |
| *Ny-Ålesund FTIR, Norway* | *78.92* | *11.92* | *-15.5* | *-5.5* |
| *Thule FTIR, Greenland* | *76.53* | *-68.74* | *-9.3* | *-3.2* |
| Resolute, Canada | 74.72 | -94.98 | N/A | -4.5 |
| Scoresbysund, Greenland | 70.48 | -21.95 | -22.9 | -4.4 |
| *Kiruna FTIR, Sweden* | *67.41* | *20.41* | *-4.1* | *-4.1* |
| Sodankylä, Finland | 67.36 | 26.63 | -12.2 | -4.2 |
| Lerwick, United Kingdom | 60.13 | -1.18 | -8.0 | -2.6 |
| Churchill, Canada | 58.74 | -93.82 | N/A | -2.4 |
| Edmonton, Canada | 53.55 | -114.10 | N/A | -0.2 |
| Goose Bay, Canada | 53.29 | -60.39 | N/A | -0.7 |
| *Bremen FTIR, Germany* | *53.13* | *8.85* | *-8.2* | *-1.3* |
| Legionowo, Poland | 52.40 | 20.97 | -5.8 | -2.6 |
| Lindenberg, Germany | 52.22 | 14.12 | -11.1 | -2.3 |
| DeBilt, Netherlands | 52.10 | 5.18 | -6.0 | -0.9 |
| Valentia, Ireland | 51.94 | -10.25 | -5.5 | -0.5 |
| Uccle, Belgium | 50.80 | 4.36 | -6.6 | -0.4 |
| Hohenpeissenberg, Germany | 47.80 | 11.01 | -10.3 | -0.6 |
| *Zugspitze FTIR, Germany* | *47.42* | *10.98* | *-8.1* | *0.3* |
| *Jungfraujoch FTIR, Switzerland* | *46.55* | *7.98* | *-5.7* | *3.9* |
| Payerne, Switzerland | 46.81 | 6.94 | -10.2 | 0.2 |
| Haute Provence, France | 43.92 | 5.71 | -5.1 | -0.5 |
| *Haute Provence LIDAR, France* | *43.92* | *5.71* | *-1.6* | *-0.5* |
| *Toronto FTIR, Canada* | *43.66* | *-79.40* | *-4.9* | *-0.1* |
| Trinidad Head, California, USA | 41.05 | -124.15 | -12.0 | -1.3 |
| Madrid, Spain | 40.45 | -3.72 | -6.3 | 0.4 |
| Boulder, Colorado, USA | 39.99 | -105.26 | -4.3 | 7.8 |
| *Boulder FTIR, Colorado, USA* | *39.99* | *-105.26* | *-9.8* | *7.8* |
| Tateno (Tsukuba), Japan | 36.05 | 140.13 | -3.6 | 0.5 |
| *Table Mountain LIDAR, California, USA* | *34.40* | *-117.70* | *-2.6* | *4.7* |
| Izana, Tenerife, Spain | 28.41 | -16.53 | -1.6 | 0.0 |
| *Izana FTIR, Tenerife, Spain* | *28.30* | *-16.48* | *-6.3* | *0.0* |
| Hong Kong, China | 22.31 | 114.17 | 0.0 | 3.2 |
| Hilo, Hawaii, USA | 19.72 | -155.07 | -1.7 | 5.6 |
| *Mauna Loa FTIR, Hawaii, USA* | *19.54* | *-155.58* | *N/A* | *5.6* |
| **Northern extratropical station average ±standard deviation** | 50.94 ±16.98 | -29.57 ±66.63 | **-7.3 ±4.6** | **-0.5 ±3.6** |
| Paramaribo, Suriname | 5.81 | -55.21 | -1.0 | 3.6 |
| Pago Pago, American Samoa | -14.25 | -170.56 | -10.8 | -3.0 |
| Suva, Fiji | -18.13 | 178.32 | -5.8 | -5.2 |
| *Wollongong FTIR, Australia* | *-34.41* | *150.88* | *0.3* | *0.8* |
| Broadmeadows, Australia | -37.69 | 144.95 | 1.3 | 2.3 |
| Lauder, New Zealand | -45.04 | 169.68 | -1.4 | 1.4 |
| *Lauder FTIR, New Zealand* | *-45.04* | *169.68* | *3.7* | *1.4* |
| Macquarie Island, Australia | -54.50 | 158.94 | 1.7 | 3.0 |
| **Tropical and Southern Hemisphere station average ±standard deviation** | -30.41 ±20.00 | 93.33 ±131.40 | **-1.5 ±4.7** | **0.5 ±3.1** |

Table S1. Similar to Table 1, but showing the average (April to August, 1 to 8 km) tropospheric ozone anomaly observed in 2020 at each station, and simulated at the CAMS grid-point next to the station. Two additional rows (bold-face) show the 2020 tropospheric anomaly averaged over all northern extratropical stations, and averaged over tropical and Southern Hemisphere stations.
